# Full-Scale of a Compost Process Using Swine Manure, Human Feces, and Rice Straw as Feedstock

**DOI:** 10.3389/fbioe.2022.928032

**Published:** 2022-07-01

**Authors:** Yi Gao, Chunxue Zhang, Lu Tan, Xiaocheng Wei, Qian Li, Xiangqun Zheng, Fang Liu, Jiarui Wang, Yan Xu

**Affiliations:** Agro-Environmental Protection Institute, Ministry of Agriculture and Rural Affairs, Tianjin, China

**Keywords:** rural waste, mixed compost, compost maturity, microbial succession, composting effect

## Abstract

Regarding the composting of rural waste, numerous studies either addressed the composting of a single waste component or were conducted at a laboratory/pilot scale. However, far less is known about the mixed composting effect of multi-component rural waste on a large scale. Here, we examined nutrient transformation, maturity degree of decomposition, and succession of microbial communities in large-scale (1,000 kg mixed waste) compost of multi-component wastes previously optimized by response models. The results showed that multi-component compost can achieve the requirement of maturity and exhibit a higher nutritional value in actual compost. It is worth noting that the mixed compost effectively removed pathogenic fungi, in which almost no pathogenic fungi were detected, and only two pathogenic bacteria regrown in the cooling and maturation stages. Structural equation models revealed that the maturity (germination index and the ratio of ammonium to nitrate) of the product was directly influenced by compost properties (electrical conductivity, pH, total organic carbon, moisture, temperature, and total nitrogen) compared with enzymes (cellulase, urease, and polyphenol oxidase) and microbial communities. Moreover, higher contents of total phosphorus, nitrate-nitrogen, and total potassium were conducive to improving compost maturity, whereas relatively lower values of moisture and pH were more advantageous. In addition, compost properties manifested a remarkable indirect effect on maturity by affecting the fungal community (*Penicillium* and *Mycothermus*). Collectively, this evidence implies that mixed compost of multi-component rural waste is feasible, and its efficacy can be applied in practical applications. This study provides a solution for the comprehensive treatment and utilization of rural waste.

## 1 Introduction

According to statistics, global rural waste generation reached 9.05 billion tons as of 2018, at least one-third of which is not properly treated (Qu et al., 2020). Solid waste pollution poses a serious threat to the environment and human health, as a result of either direct emission or inappropriate treatment (Diener et al., 2011). Moreover, rural waste often cannot be not completely treated, which causes secondary pollution in the receiving environment. At present, the cases of compost used in the treatment of livestock manure, domestic waste, and straw, etc. have been widely documented (Wang et al., 2020; Wang et al., 2021). As previously reported, single component compost presented a series of drawbacks, such as high moisture content, low degradation rate, low carbon-to-nitrogen ratio, small particle size, and high viscosity, etc. (Zhu, 2007; Bernal et al., 2009). Then, an increasing number of studies have been conducted on co-composting to improve composting quality by adding amendments. For instance, the addition of straw and tail vegetables can effectively reduce the pathogenic factors in pure manure compost (Li H. et al., 2021). Hence, the mixed composting of rural wastes has gradually developed into a solution that cannot just increase the waste disposal rate but also improve the treatment effect. In addition, the Chinese government announced the “*Five-Year Action Plan for Elevating Improvement of Rural Living Environment (2021–2025)*” in 2021, which clearly noted the need to promote the resource treatment and use of rural domestic waste, human feces, and agricultural production of organic waste.

Even so, most studies have been confined to the co-processing of up to two components. Qian et al. (2014) performed a pilot-scale study to determine the effects of the mixed composting process of poultry manure and rice straw. Wang et al. (2020) explored the functionality of the fungal community during the large scale aerobic co-composting process of swine manure and rice straw. However, little information is available on composting with multi-component rural waste. Previously, we optimized the best proportion of swine manure, human feces, rice straw, and kitchen waste through small-scale tests and mixed models, acquiring an optimal mixed compost proportion of 41.4% swine manure, 13.7% human feces, and 44.9% rice straw (Gao et al., 2021). Regrettably, the optimal composite composting ratio was determined using only NI (NH_4_^+^-N/NO_3_^−^-N), seed germination index (GI), and T (the final C/N ratio/the initial C/N ratio) as response parameters, without large-scale verification. Therefore, the actual effect of composting remains unknown. As is well known, there are differences in the effect between small-scale experiments and pilot-scale trials. Semitela et al. (2019) found that a volume change in the micro-composting experiments occurred much faster than that in the pilot-scale, with a volume reduction rate approximately four times higher during the first 3 weeks. As previously stated, it was corroborated that pH, temperature, and C/N ratio had an influence on the effect of mixed composting (Zhou et al., 2019), still, most of them were in reliance on the properties of compost products or simply subjective judgment to evaluate the overall effect of the compost. The trait of substance transformation in the process of mixed composting is still vague. This will lead to the inability to optimize the effect of mixed composting in a targeted manner at a later stage. Thus, it is necessary to systematically examine the changes in biotic and abiotic factors in the whole process of mixed composting.

We developed a mixed compost of rural multi-component wastes based on a previously obtained compost ratio. The study aimed to 1) investigate the effect of large-scale mixed compost, 2) understand the dynamic changes in biotic factors, mainly enzyme activities and the microbial community during composting, and 3) dissect the key drivers of mixed compost maturity. The outcome can provide a practical technology for the efficient co-processing of rural waste.

## 2 Methods and Materials

### 2.1 Composting Process and Sampling

Composting tests were carried out at the same place where raw materials were obtained (the resource utilization center of livestock and poultry manure in Yangjiapo Town, Tianjin Binhai New area) (Supplementary Figure S1). The physicochemical properties of the raw materials are listed in Table 1. Composting was conducted in triplicate, and the dimension of each composting pile was 1.8 m × 1.2 m × 1.5 m (length × width × height). Before each test, dried straw was mechanically shredded into small particles of sizes smaller than a 25-mesh. The raw material ratio of compost referred to the optimal ratio in the previous study (Gao et al., 2021), in which the ratio of swine manure, human feces, and rice straw were 0.40: 0.15: 0.45. The initial moisture content of the mixed raw material is set at about 60 wt% and the turning frequency of compost is referred to in the study by Xu et al. (2022).

**TABLE 1:** Physicochemical characteristics of the composting materials.

Parameter	Swine manure	Human feces	Rice straw
pH	8.8 ± 0.86	8.1 ± 1.25	6.5 ± 0.58
Organic matter (%)	56.8 ± 2.12	10.2 ± 1.36	94.3 ± 5.69
Moisture content (%)	80.52 ± 1.69	60.56 ± 4.12	60.24 ± 2.58
Total organic carbon (%)	21.56 ± 2.34	6.31 ± 1.28	51.28 ± 0.59
Nitrogen content (%)	2.64 ± 0.69	0.59 ± 0.08	1.08 ± 0.36

The entire composting process lasted for 102 days, which was divided into three phases according to the temperature changes during composting, namely, the mesophilic phase (MEP: 0–7 days), thermophilic phase (THP: 8–40 days), and cooling phase (COP: 41–101 days). The samples were collected at 0 (the initial mixed compost raw material), 3, 6, 12, 25, 38, 44, 63, 82, and 101 days. One aliquot was dry-preserved at −80 °C for subsequent DNA extraction and the others were stored at 4 °C for analyzing composting properties.

### 2.2 Physicochemical Analysis

The extracts of the fresh composting samples (1:10, w/v, sample/deionized water) were used to determine the electrical conductivity (EC) by using a conductometer (DJS-1C, Shanghai, China). The total phosphorus (TP) was measured using a chemical analyzer (CleverChem 380) and total potassium (TK) was determined by using an atomic absorption spectroscope (Agilent AA240). The elemental analyzer (VARIO EL III) was used to quantify the total carbon (TC) and total nitrogen (TN) concentration. Moisture and organic matter (OM) depend on drying at 105°C (24 h) and combustion at 500°C (5 h) to determine final values, respectively. Seed germination (GI) tests were conducted using extract solutions prepared by extraction of the compost in H_2_O (1:10, w/v), centrifugation at 4500 rpm for 10 min, followed by filtration through a 0.45 μm membrane. GI was calculated according to the equation [GI (%) = (A1 × A2)/(B1 × B2) × 100%], where A1 and B1 represent the germinated seed numbers in extract-treated and control dishes, respectively, and A2 and B2 represent the average root lengths of extract-treated and control seeds, respectively. The samples for the measurement of ammonium (NH_4_^+^-N) and nitrate (NO_3_^−^-N) were extracted with 0.01 M CaCl_2_ solution, then shaken at 150 rpm for 1 h, and finally determined by using the flow-injection analyzer (SEAL Analytical, Germany) (Li et al., 2022).

### 2.3 Enzyme Activities

The kit used 3,5-dinitrosalicylic acid to react with the end product reducing sugar to produce a brownish-red substance with a characteristic absorption peak at 540 nm, which leads to cellulase activity. The urease was analyzed by the colorimetric method of indophenol blue, which has the maximum light absorption at 578 nm, and its color depth was proportional to the ammonium nitrogen content in the solution, and then the sample urease activity size was calculated. The enzyme activity of polyphenol oxidase was determined following the protocols described in the studies by Zeng et al. (2010). Three kits were purchased from Suzhou Grace Biotechnology Co., Ltd. (Suzhou, China).

### 2.4 Microbial Community Analysis

Total genomic DNA samples were extracted using the OMEGA Soil DNA Kit (M5635-02) (Omega Bio-Tek, Norcross, GA, United States), according to the manufacturer’s instructions. The quantity and quality of the extracted DNAs were measured by using a NanoDrop NC2000 spectrophotometer (Thermo Fisher Scientific, Waltham, MA, United States) and by agarose gel electrophoresis, respectively. The primers 338F (5′-ACT​CCT​ACG​GGA​GGC​AGC​A-3′) and 806R (5′-GGACTACHVGGGTWTCTAAT-3′) were used to amplify the V3-V4 region of the bacterial 16SrRNA, and the ITS-V1 primer was used to amplify the fungal ITS region (sample-specific 7-bp barcodes were incorporated into the primers for multiplex sequencing). Vazyme V AHTSTM DNA Clean Beads (Vazyme, Nanjing, China) and Quant-iT PicoGreen dsDNA Assay Kit (Invitrogen, Carlsbad, CA, United States) were used to purify and quantify the PCR amplicons. Subsequently, equal amounts of amplicons were combined, and paired-end 2 × 250 bp sequencing was performed using the Illumina MiSeq platform with a MiSeq Reagent Kit V3 at Shanghai Personal Biotechnology Co., Ltd. (Shanghai, China).

### 2.5 Statistical Analysis

All statistical analyses and mapping were performed using Excel 2016 and Origin 2020 software packages. One-way analysis of variance was carried out to analyze the physicochemical data as the means of three replicates, and the Duncan test assisted in separating the means (*p* < 0.05). Gephi software was used for network visualization with a calculation of topological properties. We generated structural equation models (SEMs) to evaluate the contribution of composting properties, enzyme activity, bacterial community, and fungal community to the compost maturity with the smart PLS 3 software.

## 3 Results and Discussion

### 3.1 The Trait of Abiotic Factors During Composting

#### 3.1.1 Physical Properties: Temperature, pH, and Electrical Conductivity

**FIGURE 1:**
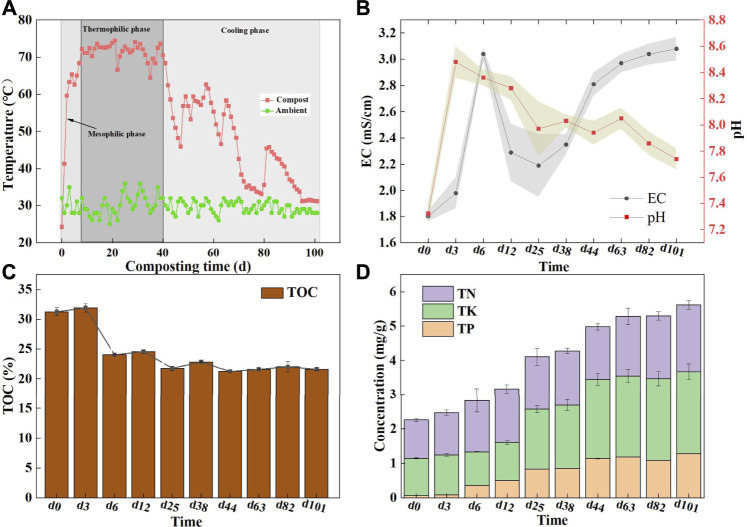
Changes of physicochemical parameters during composting; **(A)** Temperature during co-compost process; **(B)** pH and EC, the stripe represents the standard deviation; **(C)** The concentration of total organic carbon (TOC) and **(D)** The concentrations of TN, TP and TK.

Temperature is deemed as a primary indicator of compost since it is closely related to organic matter decomposition and microorganism growth and microbial communities (Wei et al., 2022). It was observed that the mixed composting temperature rose rapidly in the first 2 days and reached the maximum temperature of 74.5°C on the 7^th^ day (Figure 1A), moreover, can last for 33 days with the temperature of more than 70°C. It was demonstrated that multi-component composting enabled the rapid increase in the temperature and prolong the thermophilic phase, as compared to single-component or two-component compost (Wang et al., 2020). This implied that mixed composting could present higher fermentation efficiency with a more adequate fermentation process. As is known, the high temperature can effectively eliminate pathogens and weed seeds in the compost. After 41 days, the compost temperature gradually declined, such a fluctuation was primarily owed to the more sufficient mixing of microorganisms and materials after turning the pile, thereby enhancing the biochemical degradation of materials.

pH is an important condition in relation to the success of composting, which is associated with the growth, reproduction, and physiological metabolism of microorganisms in the compost. Here, pH manifested an alkaline with a range of 7.32–8.48 in composting. Previous studies noted that the pH in the range of 6.7–9.0 well supported microbial activity during composting (Meng et al., 2020). As a result, the pH of the whole process was appropriate. When the composting entered the thermophilic stage, the pH showed a descending trend, mainly owing to the high temperature caused by NH_4_^+^-N volatilizing to NH_3_, while thermophilic microbes work diligently to decompose organic matter and produce small molecule acids. That was also supported by previous studies (Wang et al., 2020). The pH dropped to 7.74 at the cooling phase, probably due to the degradation of organic matter which led to the release of CO_2_ and organic acids; similarly, the study by Wang et al. (2021) also rendered a similar observation.

The EC as an essential index can reflect the change in soluble salt contents upon the composting process. Admittedly, high EC in the compost is not advisable since salt is phytotoxic and negatively affects crop production (Meng et al., 2020). It can be seen here that after 6 days of composting, the EC rapidly rose to 3.04 mS/cm **(** Figure 1B), which was attributed to water loss caused by evaporation and a net loss of dry matter caused by the decomposition of organic matter, which was similar to previous studies (Silva et al., 2009). After that, the EC declined slightly, as a result of the loss of some salt ions in the leachate. In the cooling phase, the organic matter was further decomposed, resulting in a decrease in the total weight, while the salt was residual in the compost, resulting in the gradual increase of the EC content, and eventually stabilized at 3 mS/cm. It is worth affirming that the safe threshold of 4 mS/cm has never been exceeded throughout the composting process (Awasthi et al., 2018).

#### 3.1.2 Chemical Properties: Nutrients

Changes in the TOC, TN, TP, and TK concentrations were tracked to reflect nutrient conversion across the mixed composting process. As an extremely important carbon and energy source for microorganisms, the TOC is involved in several complex microbial metabolic processes to the extent which reflects the quality of the compost. As shown in Figure 1C, the decline in the TOC content mainly occurred in the initial stage of composting with a decrease of 7.23%. This loss was higher than that in a previous report showing a TOC loss of approximately 4% during the initial 5–6 days in livestock manure composting (Li M.-X. et al., 2021), potentially owing to the high availability of easily degradable organics in a multi-component compost, which accelerated the decomposition rate of the TOC. After composting, the organic matter content was uncovered at 37.24%, which was higher than the Chinese national organic fertilizer standard of 30% (NY 525/T-2021). It is thus believed that the mixed compost of rural multi-component wastes can yield high-quality organic fertilizer. As composting progressed, the relative content of the TN gradually increased from 1.11 to 1.95%, which may have been caused by the massive degradation of carbon-containing compounds (Castaldi et al., 2008). Similar to the TN variance, the TP exhibited an ascending trend from 0.07 to 1.28% during composting, which was consistent with the result of Wei et al. (2015), who showed that the proportion of phosphorus in the final product was higher than that in the early stages of composting. Similarly, the TK content showed an overall upward trend, with a minimum of 0.93% on the 6th day and a peak of 2.39% on the 101^st^ day **(** Figure 1D). The increase in the TP and TK was attributed to the “concentration effect,” in which the organic matter was lost in the form of CO_2_, NH_3_, and H_2_O, while the TP and TK were still retained in the compost (Wei et al., 2022). As the aforementioned evidence, the fermentation and decomposition capacity of the mixed compost is superior to that of one- or two-component compost. This may be ascribed to the high bioavailability of organic matter in the composite raw materials. Also, multi-component compost can preserve more nutrient elements that enable the production of higher-quality organic fertilizer products. This will be an advantage of multi-component composting.

### 3.2 Comprehensive Evaluation of Composting Effects

**FIGURE 2:**
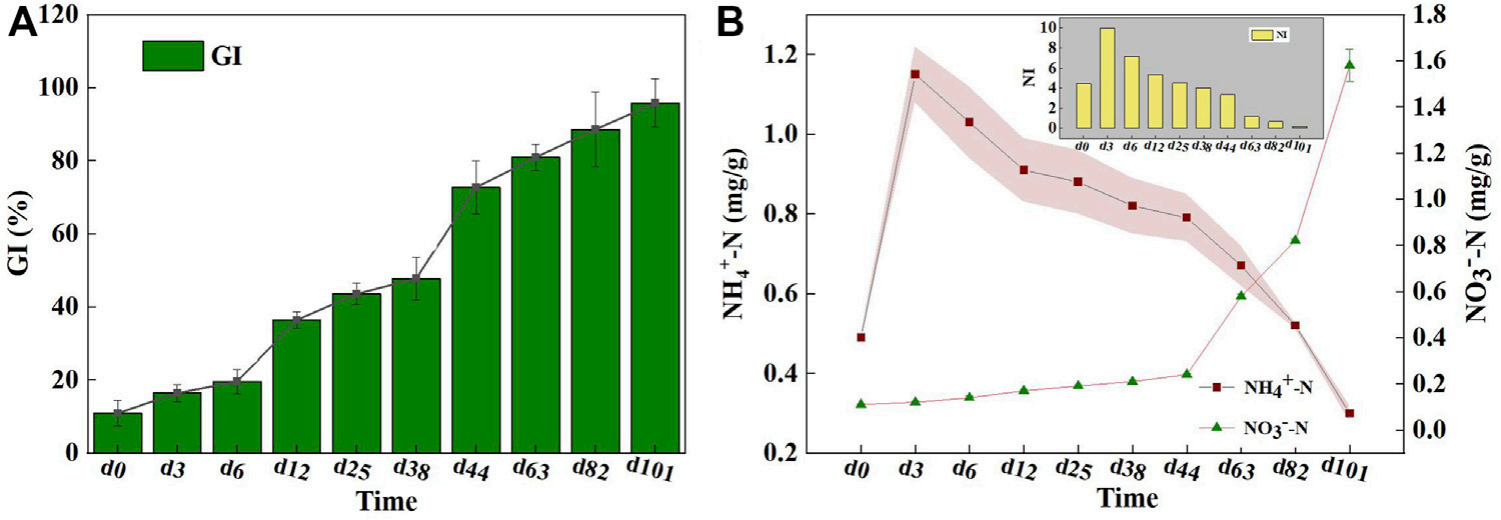
The changes of **(A)** GI and **(B)** NH_4_^+^-N/NO_3_^−^-N and NI during composting. The stripe represents the standard deviation.

The quality of final products under large-scale composting should be systematically evaluated using the aforementioned characteristics, and the maturity indexes including the germination index (GI) and NI (NH_4_^+^-N/NO_3_^−^-N), both of which were commonly used to assess compost safety (Gao et al., 2021). There was a remarkable increase in the GI, ranging from 10.90–95.80% during composting **(** Figure 2A). The compost was considered phytotoxin-free when the GI value exceed 80% (Zhou et al., 2019); thus, it was determined that the mixed compost product met the requirement of maturity according to the organic fertilizer standard.

Figure 2B showed the dynamic changes in NH_4_^+^-N, NO_3_^−^-N, and the NI during the composting process. In the early period of composting, the NH_4_^+^-N content increased rapidly from 0.49 to 4.25 g/kg, probably because of the conversion of organic-N into NH_4_^+^-N by ammonification (Meng et al., 2020). Still, the NO_3_^−^-N amounts remained relatively low during the first 44 days of composting. Indeed, NO_3_^−^-N formation from nitrification was confined as a result of the activity and growth of ammonia oxidizers, which were likely to be inhibited by the higher temperature and paucity of dissolved oxygen caused by intensive organic matter degradation (Gao et al., 2010). A marked increase in the NO_3_^−^-N content was observed as the cooling phase started. Compared with NH_4_^+^-N, NO_3_^−^-N is more easily absorbed and used by plants, which denoted that the high-quality compost organic fertilizer should yield sufficient NO_3_^−^-N (Gross et al., 2011). Also, the highest NI was acquired in the first 3 days of composting, then it gradually dropped to 0.19 until the end of composting. Referring to the relevant standards of the California Compost Quality Council, NI lower than 0.5 after composting can be regarded as very mature, whereas 0.5–3.0 is considered mature (Dahiya et al., 2018). It is thus considered that the end-product was mature, meaning that the mixed compost can accord to the availability of organic products.

Overall, the indexes required by the Organic Fertilizer Standard (NY 525/T-2021) involving pH, moisture content, organic matter, and total nutrient (listed in Table 2) should all be met as a prerequisite. It can be seen here that, multi-component compost products had achieved complete maturity and possessed a higher nutritional level. As previously reported, although composting of rural household waste alone can meet the maturity requirement, it showed lower nutrient levels and higher relative abundances of pathogenic bacteria in the final product (Muscolo et al., 2018). The multi-component composts in other studies albeit meet the standard requirements, had complex composting processes, and large investments (Table 2). Collectively, this large-scale mixed composting can be used in practice as a solution to improve rural waste disposal.

**TABLE 2:** Quality analysis of compost products.

Substrate	Index					Composting technology	Reference
	Organic matter (based on dry weight), %	Total nutrient contents (based on dry weight), %	Moisture mass fraction (fresh sample), %	pH	GI (%)		
-	≥30	≥4.0	≤30	5.5–8.5	≥70	-	NY 525/T-2021
Swine manure, human feces, and rice straw	37.24	5.62	28.82	7.74	95.8	Windrow composting	This study
Swine manure, saw dust, and rice husk	62.51	9.59	21.92	8.32	-	A 40-m^3^ composting reactor	Liu et al. (2020)
Blue-green algae sludge, livestock feces, and straw	57.91	6.59	28.36	8.00	114.5	The device included a main composting vessel, feeding and discharge conveyors, and a gas removal biofilter system	Zhang et al. (2021)
Fresh swine manure, composted swine manure, and maize straw	49.48	8.4	32	8.22	71	A patent compost tray	Wei et al. (2022)

-: unknown.

### 3.3 The Biotic Factors Involved in Composting

#### 3.3.1 Enzymatic Activities

**FIGURE 3:**
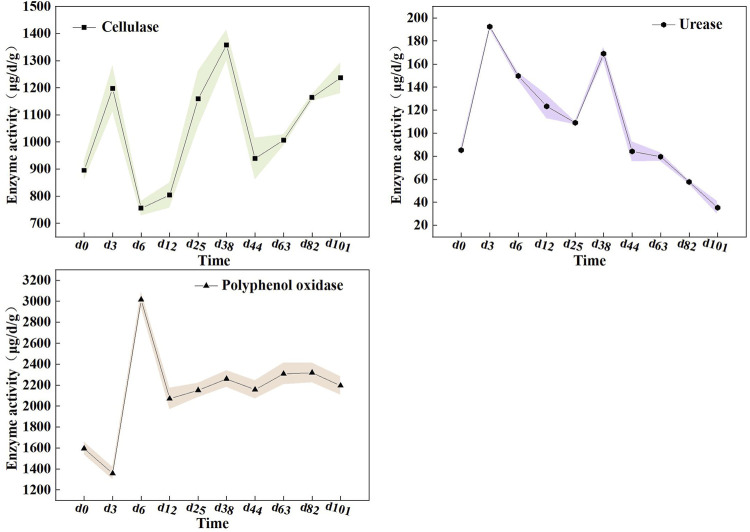
The activities of emzymes during composting. (a) cellulase, (b) urease and (c) polyphenol oxidase.

Microorganisms metabolize insoluble components by secreting various enzymes, which can characterize the degrading capacity of the substrates (Kong et al., 2018). Herein, we tracked enzyme changes to reflect the transformation of organic matter during the composting process (Figure 3). The cellulase activity is often used to indicate the degradation of cellulose in a material during composting (Zhang and Sun, 2014). It was observed that the cellulase activity peaked during the thermophilic phase, up to 1,357.71 μg/d/g, which was in agreement with previous results about co-composting of pig manure and straw (Li et al., 2020). Unexpectedly, the cellulase activity remained at a high level during the cooling phase of the compost, which was different from the traditional compost that exhibited a lower cellulase activity during this phase. As Du et al. (2019) reported, in the heating and thermophilic stages, it generally decomposes easily degraded organic matter, whereas the cooling stage mainly tackled cellulose, which is difficult to degrade by microorganisms. Hence, we consider that the mixed compost can promote the decomposition of cellulose, as evidenced by the high cellulose enzyme activity across the composting process, especially in the presence of high cellulose substances, such as straw. This will be conducive for improving the efficiency of aerobic fermentation.

In addition, the results showed that urease activity peaked on the 3rd day with a maximum level of 192.43 μg/d/g. This observation was similar to the profile reported by Wang et al. (2020). As is known, urease catalyzes the conversion of urea into ammonia, which plays an important role in waste decomposition. Lower urease activity was potentially related to the degradation of available nitrogen compounds, which was also the primary cause of the persisting declination of the ammonia nitrogen content in the later stage of composting. Nevertheless, urease activity exhibited a remarkable upward trend in the thermophilic period, unlike previous studies showing a consistent decline from the peak (Li et al., 2020). Theoretically, multi-component compost was endowed with a high microbial metabolism that was resistant to high temperatures, which can accelerate the mineralization and decomposition rate of nitrogen-containing organic matter. This can partly explain the elevated urease activity in the mixed compost system.

Here, we also examined polyphenol oxidase which was capable of catalyzing lignin disintegration and condensing quinines and amino acids formed in lignin oxidation into humic acids, to state the process of humification (Guo et al., 2012). The results showed that the activity of polyphenol oxidase decreased in the early stage of composting and then increased rapidly, attaining a maximum value of 3015.99 μg/d/g on the 6^th^ day. Of note, the activity of polyphenol oxidase was maintained in a high and stable range until the end of composting compared with the observation of the previous study (Zhang and Sun, 2018). It suggested that this mixed compost can develop more abundant humification.

These evidences indicated that multi-component composting could effectively improve the activities of cellulase, urease, and polyphenol oxidase enzymes which are involved in fermentation. It is speculated that the key reason was that multi-component composting presented a high microbial diversity, which can accelerate microbial metabolism to yield enzymes. To be sure, these enzymes play a critical role in the mixed composting system as they regulate the degradation of multiple components.

#### 3.3.2 Microbial Community

**FIGURE 4:**
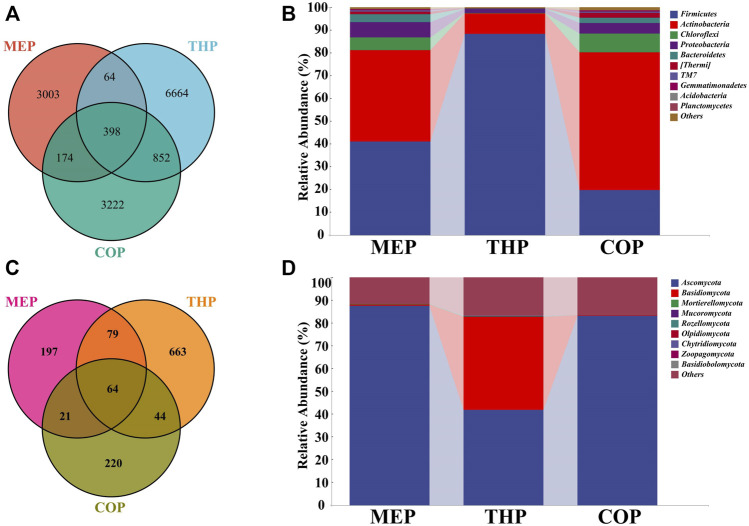
The microbiome composition during composting. **(A)** Bacterial and **(C)** fungal Venn diagram of different phases; **(B,D)** are compositions of bacterial and fungal communities during composting at the phylum-level, respectively.

Figure 4A showed the compositions of bacterial communities in the different composting phases. A total of 3,639, 7,978, and 4,646 OTUs were retrieved at MEP, THP, and COP, respectively. Apparently, the richness of the bacterial community in the multi-component compost was significantly higher than that of the two-component compost (*p* < 0.01) (Jiang et al., 2019). Also, unique OTUs, that is, non-shared OTUs were observed in each stage, especially THP, where unique OTUs accounted for 46.35% of the total OTUs, suggesting that the blooms of some specific bacteria may get involved in degrading organic matter which occurred in the thermophilic phase. To explore temporal changes in community composition, the relative abundances of bacterial taxa were compared at the phylum level. *Firmicutes*, *Actinobacteria*, *Chloroflexi*, and *Proteobacteria* were the four dominant phyla in composting **(** Figure 4B). These phyla accounted for more than 90% of the total identified sequences, which was consistent with the results of other studies (Jiang et al., 2019; Zhang et al., 2021). In the MEP of the compost, *Firmicutes* (41.03%) and *Actinobacteria* (40.21%) dominated, whereas the relative abundance of *Actinobacteria* showed a descending trend to 8.96% in the THP, and the relative abundance of *Firmicutes* increased by 88.06%. In fact, *Firmicutes* can form endospores that are highly tolerant to unfavorable conditions, allowing them to survive in unfavorable environments (Liu et al., 2018). This provided a plausible explanation for the massive proliferation of *Firmicutes* in the THP emerged. In the cooling and mature phases, *Actinobacteria* accounted for 60.47% on average, replacing *Firmicutes* as the dominant phylum, which is far higher than the 15.62% of single straw composting and 31.1% of co-composting (pig manure and straw) (Li et al., 2019; Chang et al., 2021). This benefit is reflected in the fact that more *Actinobacteria* could secrete more antibiotics to inhibit and kill pathogens during composting (Tian et al., 2013). Moreover, the abundance of *Proteobacteria* was relatively higher, up to 4.61% more than that of other conventional composts. This phylum contains diverse members, most of which exerted important roles in the carbon, sulfur, and nitrogen cycles of the compost (Zhang et al., 2021). Briefly, the relative abundances of *Firmicutes* and *Actinobacteria* in the multi-component compost were markedly higher, compared with previous compost cases, especially *Actinobacteria* which was often used as a marker for compost maturity (Jurado et al., 2014). As a consequence, multi-component composting can facilitate compost efficiency and improve maturity by increasing bacterial diversity and the dominance of functional bacteria in the materials.

The composition of the fungal community in different composting phases is shown in Figure 4C. A total of 361, 850, and 349 OTUs were acquired for the MEP, THP, and COP, respectively. This indicated that the richness of the fungal community in a multi-component compost was significantly higher than that reported in previous studies (*p* < 0.01) (Meng et al., 2018; Wang et al., 2020). As mentioned previously, fungi usually performed an important role in the degradation of lignocellulose because of the special structure of their hyphae, whereby indicating that mixed composting can be more conducive to the degradation of organic waste, as evidenced by the high richness of fungi (Duan et al., 2021). Of these, *Ascomycota* and *Basidiomycota* were recognized as the core fungi, as a result of the higher proportion of abundances in the compost (Figure 4D). This is similar to previous results (Chang et al., 2021), mostly, *Ascomycota* was detected to be the major phylum of the fungal community during composting which exerted a critical role in producing enzymes responsible for cellulose degradation (Meng et al., 2018). As well, *Basidiomycota* unveiled dominance in the decomposition and mineralization of lignocelluloses (Abdellah et al., 2021). Accordingly, the multi-component compost had a significant effect on cellulose degradation by increasing the abundance of fungal communities and promoting the activity of associated enzymes.

#### 3.3.3 Pathogens

**FIGURE 5:**
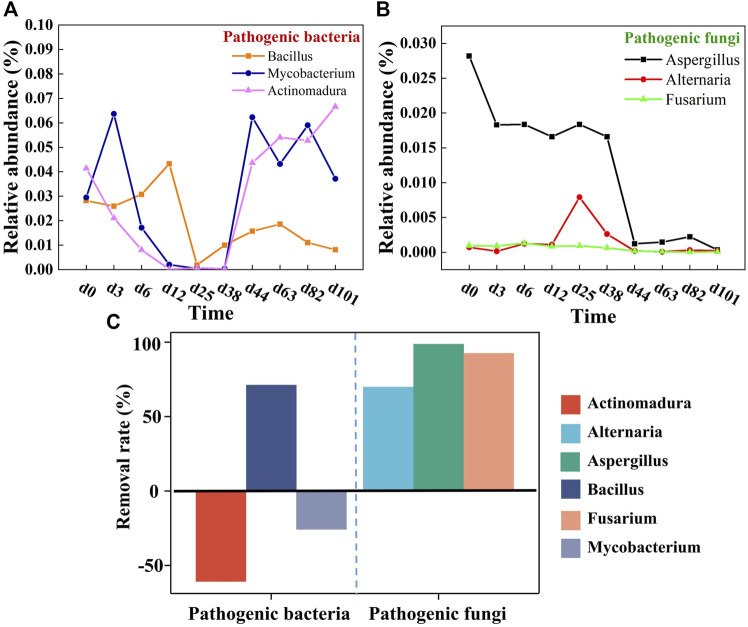
Relative abundance of **(A)** pathogenic bacteria and **(B)** pathogenic fungi in top 20 genus and **(C)** their removal rates.

The removal of pathogenic bacteria in the mixed composting system was more effective than previously reported, where a large number of pathogens still remain after composting (Xu et al., 2022). Apparently, the pathogenic bacteria *Bacillaceae-Bacillus* were significantly reduced by 71.21%. It is reported that *Bacillus* can cause an array of infections, from ear infections to meningitis, and urinary tract infections to septicemia. In most cases, they occur as secondary infections in immuno-deficient hosts or otherwise compromised hosts (Xu et al., 2022). Three pathogenic fungi, *Aspergillus*, *Alternaria,* and *Fusarium* were effectively killed, especially *Aspergillus* with a reduction of 98.76% (Figure 5). This may be related to the higher microbial diversity in the multi-component compost. Duan et al. (2021) stated that diversification of raw materials can reshape the composting micro-environment and enhance inter-microbial competitiveness, which is thus detrimental to the small population of pathogens. In addition, multi-component composting can achieve higher composting temperatures and prolong the high temperature time compared with traditional single-component/two-component composting, which in turn eliminates pathogenic bacteria more effectively (Wang et al., 2020). Even so, this system is not perfect because potential pathogens were still detected at the end of the compost. *Mycobacterium* and *Actinomadura* remained recalcitrant, possibly because of their unique cell wall structure and ability to survive some heat or chemical stressors. Once acquiring favorable conditions, it will trigger rapid reproduction (Lepesteur, 2022). Moreover, these two strains are more resistant to high temperature, whereby most of them may just be inhibited during the high-temperature period, while, with the advent of favorable survival conditions, they will regrow. It is thus considered that the next strive to extend the high-temperature period of composting will assist in the removal of pathogens (Xie et al., 2021). Of course, it is impossible and unnecessary for the compost product to be completely free of pathogenic bacteria. In most environments, such as soil, water, etc., there exist pathogenic bacteria residues. Composting is identified as successful when it effectively reduces pathogenic factors and ultimately produces a hygienic and harmless product. Overall, it can be concluded that multi-component compost features certain advantages over traditional single compost in the removal of pathogens, especially for the removal of pathogenic fungi.

### 3.4 Key Drivers of Mixed Compost Maturity

**FIGURE 6:**
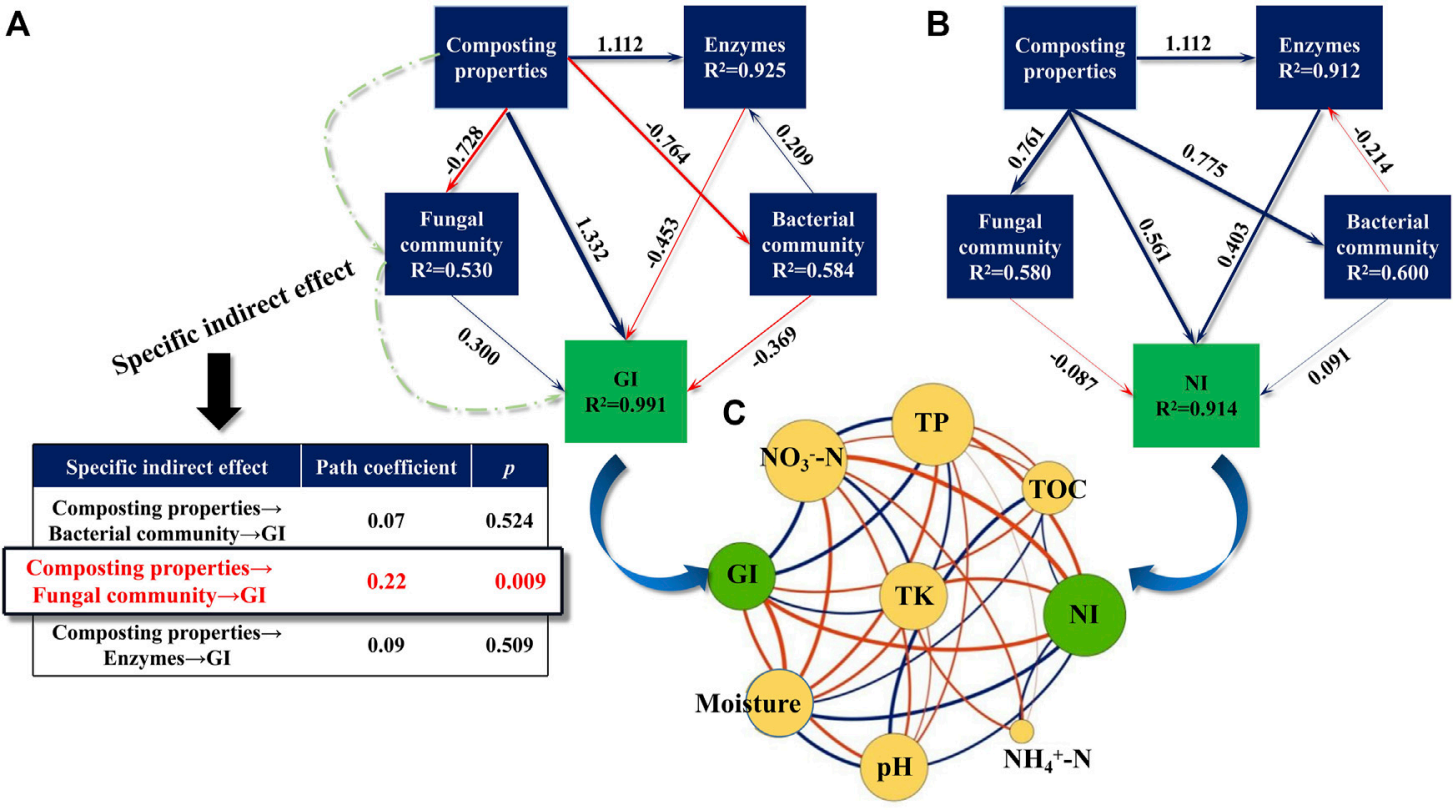
Structural equation models (SEMs) showing the pathways **(A,B)** of the different factors (environmental factors, enzymes and microorganisms) on compost maturity (GI and NI). The path coefficients are adjacent to the arrows. Blue and red lines indicate the positive and negative pathway. Co-occurrence networks between maturity indexes and abiotic factors **(C)**. A connection is observed for strong (Spearman’s ρ>|0.6|) and significant (*p* < 0.05) correlations among these variables. The green nodes indicate the parameters of maturity indexes while the orange nodes indicate biotic factors. The size of nodes is proportional to the degree of connectivity. Blue links indicate negative interactions between two individual nodes, while red links indicate positive interactions.

Maturity is an important criterion that identifies the effect of composting. Herein, structural equation modeling (SEM) was conducted to assess the contribution of compost properties (EC, pH, NH_4_^+^-N, NO_3_^−^-N, TOC, moisture, temperature, TN, TP, and TK), enzymes (cellulase, urease, and polyphenol oxidase), and the microbial community to compost maturity. As shown in Figures 6A,B, GI and NI changes were well interpreted by all variables, as evidenced by high R^2^ values (0.991 and 0.914, respectively). The path coefficients of the compost properties for the GI and NI were highest at 1.332 and 0.561, respectively, implying that compost properties are the main drivers influencing the maturity of the compost. As shown in Figure 6C, the GI had a strong positive correlation with the TP, NO_3_^−^-N, and TK (*p* < 0.05), whereas it showed a negative correlation with the moisture content, pH, and TOC (*p* < 0.05), in agreement with the results of Zhang et al. (2021). In contrast, NI was positively correlated with moisture content and pH, but had negative correlations with TOC, TK and NO_3_^−^-N (*p* < 0.05). These pieces of evidence implicated that higher contents of TP, NO_3_^−^-N, and TK were conducive to improving compost maturity, whereas relatively lower values of moisture and pH were more advantageous.

Of note, there was a specific indirect effect of compost properties on the GI (path coefficient = 0.22, *p* < 0.01), as presented by the observation that compost properties had a remarkable correlation with the fungal community, and afterward, the fungal community’s impact on the GI. This was supported by previous studies showing that the composting properties regulate the product maturity by shifting fungal communities (Xie et al., 2021). Among the fungal community of the mixed compost, it is unearthed that *Penicillium* and *Mycothermus* exerted an obvious effect on GI changes (Supplementary Figure S2). Dehghani et al. (2012) also demonstrated that *Penicillium* was an important fungus for the maturation of domestic waste compost. It has been reported that *Penicillium* degrades a number of toxic exogenous compounds produced by the decomposition of various lignins, thus contributing to a final mature compost (Leitao, 2009). Also, Wang et al. (2020) noted that *Mycothermus* was the predominant fungus during the thermophilic phase of swine manure and rice straw co-composting. In a conventional composting environment, it was found that *Mycothermus* is able to produce thermo-stable lignocellulose-degrading enzymes, thus playing an important role in the degradation of lignocellulose. Thus, multi-component composting indirectly affects the GI of compost products by enhancing the relative abundance of particular fungi capable of degrading lignocellulose. Taken together, these pieces of evidence unveiled that the maturity (GI and NI) of the product was most directly influenced by compost properties rather than other drivers (enzymes and microbial community), and that the host properties showed a specific indirect effect on the GI by affecting the fungal community, of which *Penicillium* and *Mycothermus* were key fungi implicated in GI changes.

## 4 Conclusion

This study comprehensively assessed large-scale multi-component rural waste aerobic composting, which is a prolongation of the previous theoretical optimization of multi-component mixed composting. The outcome indicated that the mixed composting with an optimal ratio of 41.4% swine manure, 13.7% human feces, and 44.9% rice straw can not only achieve complete maturity of compost but also furnish relatively high nutrients of organic products. The mixed compost presented to be somewhat surprising in killing pathogenic fungi, that is, almost no potential pathogenic fungi were detected. Still, a few pathogenic bacteria can be revived during the cooling and maturation stages. Furthermore, the product maturity (GI and NI) was mainly influenced directly by compost properties, rather than enzymes and microbial community, while compost properties showed specific indirect effects on the GI by affecting fungal communities, with *Penicillium* and *Mycobacterium* being the key fungi involved in GI variation. In general, the study provided a co-composting method with strong operability and up-to-standard products, which will assist in realizing waste reduction, harmlessness, and resource utilization.

## Data Availability

The raw data supporting the conclusion of this article will be made available by the authors, without undue reservation.
