# Supplementary material for: Full-Scale of a Compost Process Using Swine Manure, Human Feces, and Rice Straw as Feedstock
Source: Front Bioeng Biotechnol. 2022 Jul 1;10:928032. doi: 10.3389/fbioe.2022.928032 (PMC9286457; doi:10.3389/fbioe.2022.928032)
Supplement: Supplementary file 1 [file DataSheet1.docx]

Supplementary Material

# Supplementary Figures


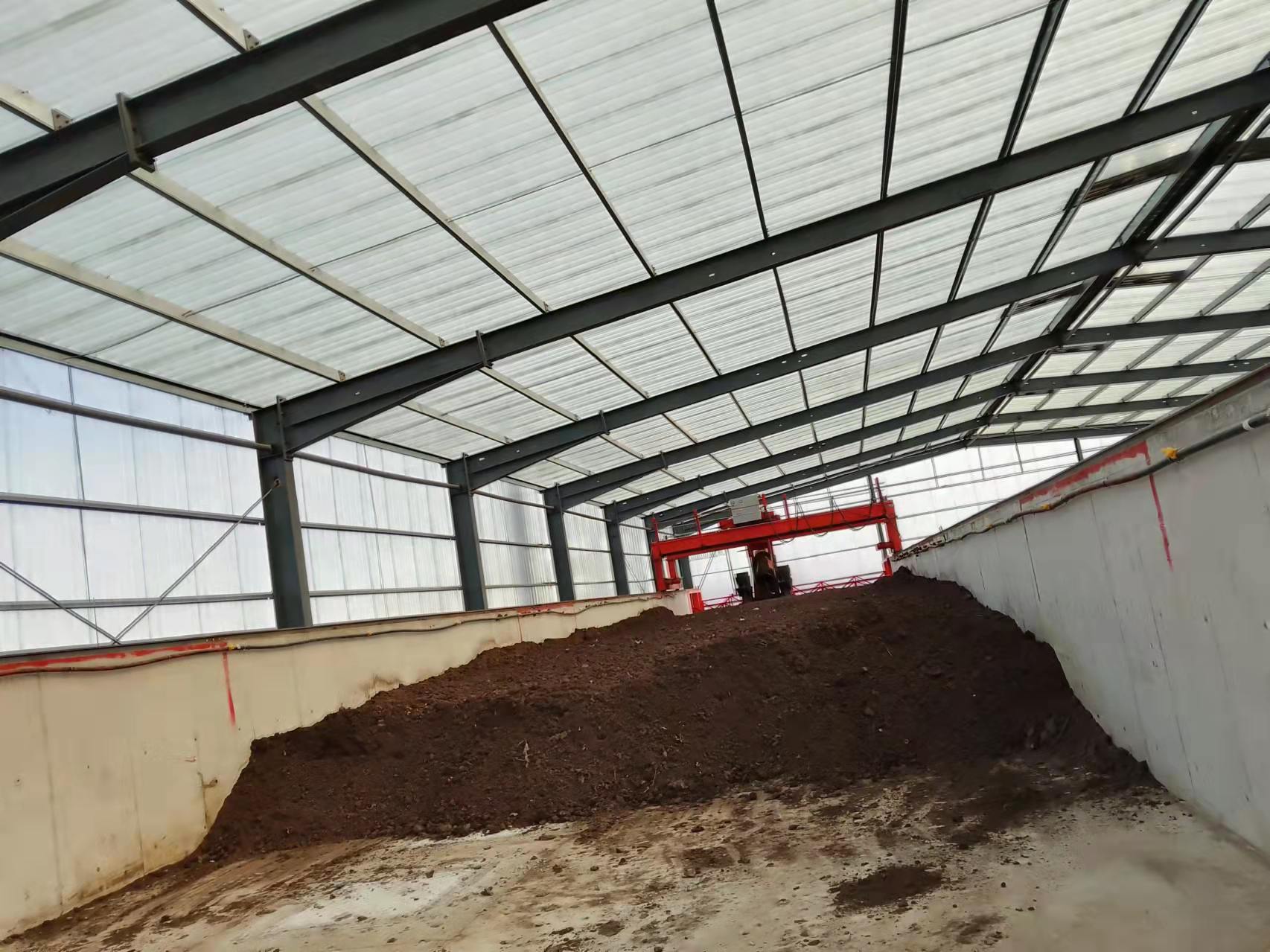


**Supplementary Figure 1.** Photo of on-site composting.


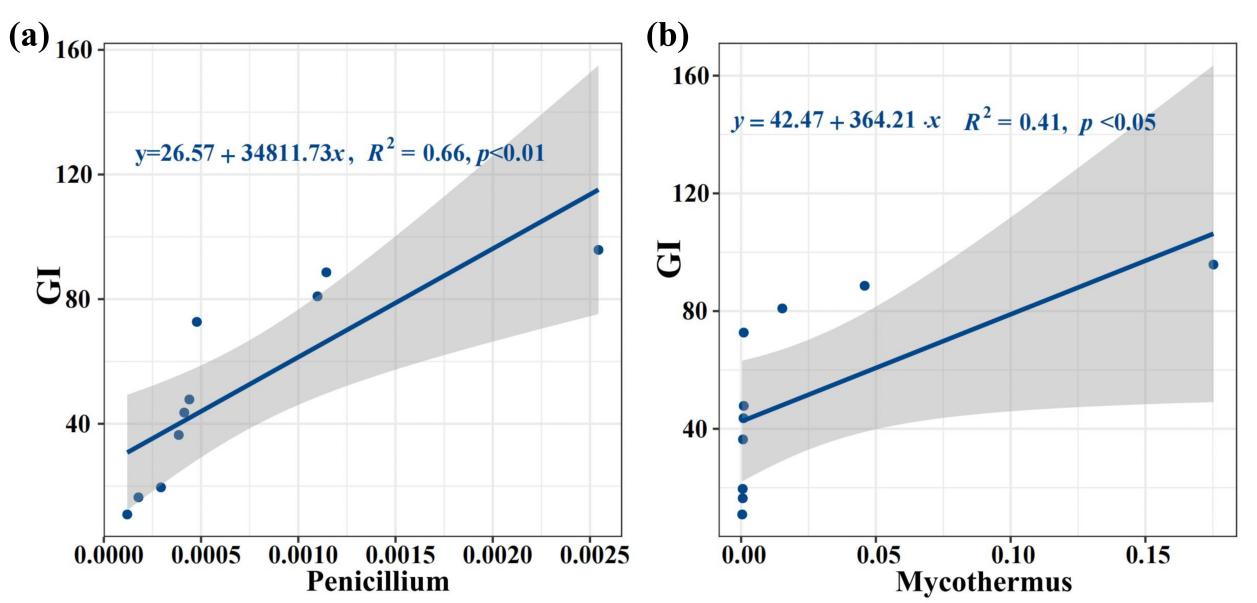


**Supplementary Figure 2.** Relationships between the *Penicillium* (a) and *Mycothermus* (b) and product maturity (GI value) during the composting.
